# Iron Deficiency Diagnosis in Sub‐Saharan Africa: Challenges, Barriers, and Opportunities

**DOI:** 10.1155/anem/9089207

**Published:** 2026-03-22

**Authors:** Bright Manu, Benedict Ofori, Abdul Rashid Kassim, Emmanuel Lante Lamptey, Kwesi Zandoh Tandoh, Kwabena Amofa Nketia Sarpong

**Affiliations:** ^1^ Department of Pathology, Microbiology, and Immunology, University of Nebraska Medical Center, Omaha, Nebraska, USA, unmc.edu; ^2^ Medical College of Georgia, Augusta University, Augusta, Georgia, USA, augusta.edu; ^3^ West African Centre for Cell Biology of Infectious Pathogens, University of Ghana, Accra, Ghana, ug.edu.gh; ^4^ West African Genetic Medicine Centre, College of Health Sciences, University of Ghana, Accra, Ghana, ug.edu.gh; ^5^ Department of Biochemistry, Cell and Molecular Biology, College of Basic and Applied Sciences, University of Ghana, Accra, Ghana, ug.edu.gh; ^6^ College of Health Sciences, Noguchi Memorial Institute for Medical Research, University of Ghana, Accra, Ghana, ug.edu.gh

**Keywords:** diagnostic challenges, inflammation and infection, iron biomarkers, iron deficiency, point-of-care testing, sub-Saharan Africa

## Abstract

Iron deficiency (ID) is the most common nutritional deficiency in sub‐Saharan Africa (SSA). The burden of ID in SSA is further amplified by high rates of infectious diseases, hemoglobinopathies, and health system challenges. Early and accurate diagnosis of ID is necessary to reduce the clinical and public health impact of ID, particularly among vulnerable groups such as children and pregnant women. Identifying ID before the onset of anemia is especially important, as anemia represents a late manifestation of depleted iron stores. Achieving earlier detection in SSA requires improved geographic and financial access to iron status testing, as well as diagnostic approaches that are appropriate for local epidemiological contexts. This review evaluates current strategies for diagnosing ID in SSA and points out key limitations that reduce their effectiveness. We emphasize that the interpretation of commonly used iron biomarkers is frequently confounded by inflammation, malaria, and inherited blood disorders, leading to misclassification and underestimation of the actual disease burden in SSA. These biological challenges are also compounded by systemic barriers, including high testing costs, limited laboratory infrastructure, reliance on distant referral facilities, and gaps in awareness at both healthcare providers and community levels. We further argue that improving ID diagnosis in SSA will require a multifaceted approach that includes the adoption of affordable, context‐appropriate diagnostic platforms and the development of regionally derived reference ranges and diagnostic decision frameworks. Such strategies would better reflect the physiological and environmental diversity across SSA and support more accurate identification of both ID with and without anemia. These efforts could provide a practical pathway toward improving early detection, guiding targeted interventions, and reducing the burden of ID across the region.

## 1. Introduction

The essential role of iron in human physiology is seen in several functions such as oxygen transport, energy production, and immunity. Inadequate levels of iron can result in fatigue, impaired growth, and increased susceptibility to infections [1]. Iron deficiency (ID) is the most common nutritional deficiency and the leading cause of anemia globally [2, 3]. Growing evidence indicates that ID on its own may play a role in the development of cardiomyopathy, even when anemia is not present, highlighting its broader systemic impact beyond hematological manifestations [4, 5]. ID is characterized by inadequate iron availability to support normal cellular and metabolic functions. The common causes of ID include inadequate intake of iron‐rich foods, particularly in individuals relying predominantly on plant‐based diets, heavy blood loss during menstruation in women, increased physiological demands during growth or pregnancy, and impaired absorption due to certain health conditions or dietary factors [6, 7].

Globally, anemia affects about one‐quarter of the population, particularly women of reproductive age and young children [8]. ID anemia (IDA) affects an estimated 1.2 billion people worldwide, while ID without anemia (IDWA), though underdiagnosed and lacking precise estimates, is believed to be at least twice as prevalent [3]. Low‐ and middle‐income countries (LMICs) bear the highest burden of anemia, with the leading causes being dietary ID, thalassemia and sickle cell disease, and malaria [9]. In sub‐Saharan Africa (SSA), these major drivers are highly prevalent. For instance, there are high rates of hemoglobin disorders such as α^+^‐thalassemia and sickle cell disease, with α^+^‐thalassemia known to directly lower hemoglobin and red cell indices while sickle cell disease modify these effects and malaria [10, 11]. Additionally, vitamin A deficiency, which impairs iron metabolism, parasitic infections, HIV/AIDS, and recent evidence of high prevalence of *Helicobacter pylori* infection further contribute to iron loss resulting in ID [12–15].

The diagnosis of ID starts with the clinical history and physical examination. This is followed by a series of laboratory tests that include a complete blood count (CBC), serum iron, transferrin, total iron‐binding capacity (TIBC), and serum ferritin levels [16]. The management of ID is challenged in clinical settings by the performance of biomarkers of iron status that are influenced by multiple factors that make interpretation difficult [17]. Due to this, the World Health Organization (WHO) recommends age‐specific ferritin thresholds to define ID, with higher cut‐offs recommended in children with inflammation to account for the effects of infection on iron markers [18]. In SSA, the high prevalence of infectious diseases and chronic inflammation can mask ID by raising ferritin and lowering serum iron and transferrin independently of actual iron stores, which can make standard diagnostic markers less reliable [19, 20]. There are also limitations in access to essential diagnostic tools and laboratory infrastructure, leading to underdiagnosis across the region [21, 22].

Beyond the effects of comorbidities and limitations in diagnostic access and laboratory capacity, geographic factors also contribute to challenges seen in the detection of ID in SSA [23]. A significant proportion of the SSA population (about 56% based on the most recent World Bank data) reside in rural and remote areas, where healthcare facilities are limited and access to the full complement of laboratory‐based iron studies is often poor [24]. These structural barriers disproportionately affect women and children and further contribute to delayed diagnosis and under‐recognition of ID across the region. In this review, we aim to reassess the diagnostic barriers to ID detection in SSA by examining how current iron status assays work, the factors that make them costly or difficult to deploy in low‐resource settings, and the reasons these limitations contribute to ID underdiagnosis. We also explore potential technical and methodological innovations, including simplified, low‐cost, and alternative diagnostic approaches, that could improve access to accurate iron assessment without compromising diagnostic performance.

## 2. Current Approaches to the Diagnosis of ID in SSA

In clinical settings, the diagnosis of ID, including both iron IDA and IDWA, relies on clinical assessment supported by hematological and biochemical biomarkers of iron status. The WHO defines ID and its severity using hemoglobin and ferritin thresholds; however, because ferritin is influenced by inflammation and infection, these thresholds remain debated across different epidemiological and clinical contexts [17, 25]. Diagnostic challenges therefore extend beyond the application of uniform thresholds, as a one‐size‐fits‐all approach may increase the risk of misclassification in populations with distinct genetic, environmental, and patterns of infectious disease burden. Various diagnostic approaches are currently accepted and employed for ID diagnosis worldwide, ranging from clinical assessments to advanced laboratory tests. Each method comes with its unique advantages and some limitations. These methods together with their advantages and limitations that have been reported recently in regard to its use in SSA are discussed below.

### 2.1. Early vs Symptom‐Based Diagnosis of ID

IDA is the most common clinical manifestation of ID and is relatively easy and inexpensive to diagnose using hemoglobin measurements [16, 20]. IDA, however, represents a late stage of ID, and relying solely on symptom‐based diagnosis misses the opportunity to intervene earlier and usually not sufficient for definitive diagnosis [26]. The lack of access to reliable diagnostics for ID in many parts of SSA causes overreliance of clinical symptoms for diagnosis. ID typically presents with signs related to reduced oxygen delivery to tissues, such as fatigue, weakness, shortness of breath, and cognitive changes [27]. Chronic anemia often develops silently, as the body gradually adapts to declining red blood cell (RBC) and hemoglobin levels [28]. Also, due to this physiological adjustment, symptoms may remain mild even in the presence of severe anemia in such cases. In contrast, acute anemia typically presents with more pronounced signs and symptoms, making timely diagnosis and intervention crucial to prevent serious complications [28]. The severity of IDA and its symptoms can vary based on the underlying cause and the impact on the body’s oxygen supply.

Although IDA is widely recognized and often diagnosed based on low hemoglobin levels, there is a growing awareness of IDWA, a condition that may present similar symptoms but is frequently overlooked in clinical settings [3]. Despite IDWA being projected to be as twice as common as IDA, it remains under‐recognized in several clinical settings, partly due to inconsistent screening guidelines [3]. Patients with IDWA may experience symptoms such as fatigue and cognitive decline despite having normal hemoglobin levels [3, 20]. Biochemical profiles typically reveal low ferritin and transferrin saturation with normal hemoglobin [29]. Some studies also suggest that, depending on the ferritin cut‐offs used, up to 72% of women may have ID [30]. These individuals often experience symptomatic improvement following iron supplementation, highlighting the clinical relevance of identifying and treating IDWA even in the absence of anemia [31]. Although IDWA can be detected through biochemical markers such as low ferritin and transferrin saturation, relying on clinical symptoms alone is insufficient. Fatigue, the main symptom of ID, is particularly difficult to interpret, as it is common in 10%–20% of primary care visits and associated with numerous other conditions, including depression, sleep disturbances, and chronic inflammation [32, 33]. Even when ferritin is used to support diagnosis, fatigue remains an unreliable indicator. For instance, ferritin levels below 6 ng/mL identified only 36% of adolescents with severe fatigue and bleeding disorders [34]. There is therefore a clear and critical need for improved, accessible, and cost‐effective diagnostic tools in SSA as reliance on symptoms alone contributes to underdiagnosis and delayed treatment.

### 2.2. Laboratory‐Based Diagnosis of ID

Laboratory testing remains the most reliable method for accurately diagnosing ID. There are global diagnostic thresholds for ID, such as those recommended by the WHO and several expert panels in different clinical settings, and while these recommendations provide a standard framework, emerging evidence indicates that these cut‐offs may not capture the true burden of ID in populations with high infection and inflammation such as in SSA. Recent multinational analyses suggest that physiologically based ferritin thresholds are substantially higher than current WHO guidelines, leading to markedly higher prevalence estimates of ID when applied across diverse populations [35]. Moreover, research in South African and Kenyan populations shows that using higher ferritin cut‐offs improves sensitivity and that locally derived, sex‐specific reference limits may be more appropriate than universal thresholds [36, 37]. These findings show that there are limitations in current global thresholds for some markers of ID and that a one‐size‐fits‐all approach complicates the efforts to properly diagnose ID in SSA and hence the need for context‐specific diagnostic standards to improve detection. The commonly used laboratory tests for diagnosing ID are discussed below, with emphasis on their practicality and relevance in SSA.

#### 2.2.1. The CBC

CBC is one of the most widely ordered diagnostic tests worldwide [38]. CBC test measures different components of the blood, but the key indicators for diagnostic purposes include hemoglobin (Hb), hematocrit (Hct), red cell distribution width (RDW), and RBC indices such as mean corpuscular volume (MCV) and mean corpuscular hemoglobin concentration (MCHC) [39]. CBC abnormalities typically reflect IDA, as IDWA often presents with normal hematologic indices and requires biochemical markers for detection. In some instances, CBC alone cannot confirm IDA without additional iron tests. Compared to more sensitive and specific tests like serum ferritin, the CBC is more affordable and widely available in resource‐limited settings such as SSA, making it a practical option for IDA. Notably, CBC has known limitations in detecting IDA because changes often appear only after anemia develops; results can overlap with other conditions like infections or thalassemia, which are common in SSA, and some patients may show normal values or misleading platelet counts [16, 40]. Since it is the most affordable among available tests, the diagnostic utility of the CBC requires validation through population‐based studies in local populations in SSA. Such studies should assess correlations between CBC indices and established biochemical markers of iron status, such as ferritin, and define context‐appropriate cut‐off values that allow reliable identification of IDA using CBC parameters without compromising diagnostic accuracy or missing earlier, nonanemic stages of ID.

Recently, machine learning models trained on large population datasets have shown promise in accurately identifying IDA based on specific CBC markers [41–44]. One study utilized data from the U.S. National Health and Nutrition Examination Survey to train a machine‐learning model that effectively distinguished IDA from other forms of anemia using RBC indices derived solely from CBC data [41]. The model demonstrated strong predictive performance, and when validated using nationwide data from Kenya, it showed comparable accuracy, highlighting its potential applicability across diverse populations [41]. Approaches such as these should be encouraged and used on large dataset generated from SSA populations that include CBC results alongside relevant confounders to evaluate how effectively CBC alone can be used to diagnose IDA in SSA since the test is widely available and more affordable than other diagnostic options.

#### 2.2.2. Serum Ferritin

Serum ferritin is a blood test that is considered the most sensitive, noninvasive way to measure the amount of ferritin, the protein that stores iron in the blood [36]. However, its accuracy can be affected in inflammatory conditions because ferritin also acts as an acute‐phase reactant and may be elevated response to inflammation independent of iron stores [35]. Therefore, serum ferritin alone cannot reliably diagnose ID when inflammation is present, and transferrin saturation is usually measured alongside it in such cases. The current WHO‐recommended threshold for ID is a ferritin concentration below 30 μg/L in children and 70 μg/L in adults with infection or inflammation, with adjustments for inflammation possible using correction factors [45]. Ferritin levels above 150 μg/L in healthy menstruating women, 200 μg/L in healthy men and nonmenstruating women, and 500 μg/L in nonhealthy adults may indicate a risk of iron overload [45]. The current threshold recommended by the WHO has, however, been questioned over the past decade, as it is considered too low and may result in many individuals with ID going undiagnosed or untreated [46]. Supporting this concern, in a recent multinational study comparing Africa, Central America, Asia, and Europe, researchers found that using physiologically based serum ferritin thresholds is more effective for detecting the clinical and functional outcomes of ID [35]. This demonstrates the immediate necessity to update current guidelines being used in health facilities to better reflect physiological differences in diverse populations like SSA.

#### 2.2.3. Transferrin Saturation, Serum Iron, and TBIC

Transferrin saturation is an important laboratory test for assessing iron status, measuring the percentage of transferrin, a plasma glycoprotein synthesized by the liver and bound to iron [47]. In cases of ID, clinicians often order this test to obtain a complete picture of a patient’s iron levels. The current threshold for transferrin saturation typically ranges from 20% to 50%, although studies have reported slight variations in these values based on sex differences [48, 49]. Levels below 20% suggest ID, while levels above the normal range may indicate iron overload in conditions such as hereditary hemochromatosis, where the body absorbs too much iron from the diet [50]. Low transferrin saturation is often accompanied by low serum iron and high TIBC, which reflects the body’s attempt to increase iron transport in response to low iron availability [51]. TIBC measures the total amount of iron‐binding sites available on transferrin and is elevated in ID and decreased in iron overload. Normal TIBC ranges are 240–450 μg/dL [52]. Low serum iron with high TIBC typically indicates ID, whereas high serum iron with low TIBC suggests iron overload. However, normal serum iron and TIBC levels do not rule out ID since iron stores can become depleted before changes appear in serum iron levels [51]. Despite their diagnostic value and the valuable information that these indices provide on iron metabolism, their routine use in SSA is constrained by diurnal variation, inflammation, and the need for fasting and multiple measurements that reduce their practicality as standalone tools in many routine clinical settings [53–55].

#### 2.2.4. Reticulocyte Hemoglobin Content

Reticulocyte hemoglobin content (CHr) measures the amount of hemoglobin in reticulocytes, which are immature RBCs [56]. CHr reflects the functional iron available for hemoglobin synthesis in reticulocytes over the previous 3–4 days and serves as an early indicator of ID before anemia develops [56, 57]. The reference range for CHr typically falls between 25 and 29 pg, although studies report slight variations in these values [58–60]. CHr is considered a highly precise test, and low levels can indicate early ID with minimal coefficient of variation [57]. It is commonly used alongside other laboratory tests to assess the cause of anemia and to guide appropriate treatment decisions. In SSA, CHr provides a more cost‐effective and sensitive diagnostic tool for detecting ID compared to traditional biochemical tests such as serum ferritin, serum iron, and transferrin saturation. Also, because CHr reflects functional iron availability prior to reductions in hemoglobin, it may be a valuable test to detect IDWA. Since CHr can be measured as part of a standard complete CBC on standard hematology analyzers, its integration into routine clinical practice in SSA is both feasible and practical. This approach could enhance the early identification and management of ID, including IDWA in the locations across SSA where standard hematology analyzers are available.

#### 2.2.5. Soluble Transferrin Receptor (sTfR)

The sTfR is not an acute‐phase reactant like ferritin, making it a more reliable marker for diagnosing ID in individuals with coexisting inflammatory conditions that may falsely elevate ferritin levels [61]. sTfR is derived from the membrane transferrin receptors on cells that require iron and reflects the amount of iron available for erythropoiesis. Elevated sTfR levels often indicate IDA. Normal sTfR values typically range from 2 to 5 mg/L in adults, while pediatric studies report lower reference ranges, generally between 1 and 3 mg/L [62]. While sTfR testing is relatively more expensive than other iron status tests, its high sensitivity, specificity, and ability to detect ID make it a valuable tool to improve diagnostic accuracy in SSA [63].

#### 2.2.6. Hepcidin Levels

Hepcidin controls iron levels by binding to ferroportin, the iron transporter on cell membranes, leading to its degradation [64]. This action reduces iron absorption from the intestine and limits iron release from stores, thereby decreasing serum iron levels [65]. In IDA, hepcidin levels are typically low or suppressed, allowing for increased iron absorption and mobilization from stores [66]. This suppression is a physiological response to ensure adequate iron availability for erythropoiesis. In contrast, hepcidin levels are often elevated in anemia of chronic disease (ACD) due to inflammation [67]. Elevated hepcidin restricts iron absorption and release, leading to iron‐restricted erythropoiesis. Thus, measuring hepcidin can help differentiate between IDA and ACD. Conditions such as infections, autoimmune diseases, and chronic inflammatory states can increase hepcidin production, leading to decreased serum iron and potential anemia [68]. In the context of SSA, hepcidin measurement may be of a great value in differentiating IDA or IDWA from anemia driven by inflammation or infection. However, widespread use is limited by the high cost, limited availability of standardized assays, and interassay variability. Even without anemia, hepcidin serves as a sensitive indicator of ID. Declines in hepcidin, alongside reductions in transferrin saturation and ferritin, can signal early ID before changes in hemoglobin or hematocrit are detectable [67]. Additionally, hepcidin can be measured in urine, allowing for noninvasive sample collection, which is particularly advantageous for infants and young children. While promising as a diagnostic tool for early ID, including IDWA, hepcidin measurement currently remains more practical in research or well‐resourced settings rather than routine clinical practice across SSA.

## 3. Challenges in ID Testing in SSA

The burden of ID in SSA is characterized by diagnostic challenges that are mainly driven by economic and infrastructural barriers. As the region with the highest global population of the most vulnerable groups affected by ID, addressing ID is important. The key challenges include limited access to diagnostic tools due to weak health infrastructure and high testing costs. In addition to factors discussed above, the lack of regular calibration of diagnostic equipment in the region complicates the interpretation of diagnostic tests.

### 3.1. Diagnostic Limitations of Iron Biomarkers

There is no single universally accepted test that can definitively diagnose or exclude ID because the condition manifests in complex and variable ways depending on the underlying causes [16]. As a result, biomarkers for assessing ID often require careful interpretation and, in many cases, must be used in combination to improve diagnostic accuracy. One major challenge is that ferritin, one of the most commonly used markers of iron status due to its sensitivity, can be elevated during inflammation, which can then cause masking of ID. To address this, regression‐correction methods have been developed to adjust iron biomarkers using inflammation markers such as C‐reactive protein (CRP) and alpha‐1‐acid glycoprotein [69, 70]. These methods provide more accurate estimates of ID, yet they are often impractical for routine clinical use due to their complexity and cost [71]. Using these adjustments, studies in high‐inflammation and malaria‐endemic settings have shown that WHO‐defined ID prevalence may be substantially underestimated; for instance, there is 31% versus 52% when corrected with regression models [72]. In SSA countries like Uganda and Kenya, the impact of malaria on iron biomarkers and immune responses varies geographically, showing the importance of context‐specific diagnostic thresholds [72]. Developing such tailored strategies can help improve detection and reduce the burden of ID across diverse epidemiological settings.

The complexity of physiological processes in the human body with regard to blood, as it is controlled by dynamic interactions between iron homeostasis, erythropoiesis, inflammation, and plasma volume shifts, makes the standardization and interpretation of hematological and iron biomarkers challenging. In addition to the impacts of biological and inflammatory confounders mentioned earlier, the effects of preanalytical physiological factors can also influence how key hematological and iron markers are interpreted. A 2021 study conducted in Ghana found that in warm climates, plasma volume contraction can cause high red cell counts, with poor agreement between hemoglobin and hematocrit, complicating accurate anemia diagnosis [73]. Clinically, the transition from a supine to seated or standing posture has been linked to hemoconcentration and elevated results, which might not accurately reflect the true iron status of the patient. It has therefore been suggested that before blood samples are taken, patients remain in supine position for at least 15 min. This is a common practice among laboratorians, but the lack of adherence to this that normally arises from poor laboratory practice should be minimized to control for misclassification of anemia status, particularly in borderline cases.

sTfR levels, known to be less affected by inflammation compared to other iron markers, can still be elevated in certain conditions. A study in Congolese children found that sTfR concentrations were significantly higher in those with glucose‐6‐phosphate dehydrogenase variants, but not in those with sickle cell variants or α‐thalassemia [74]. Additionally, sTfR levels may also rise in the presence of malaria, even in asymptomatic individuals [75]. Studies have shown that people with α‐thalassemia trait show lower Hb, hematocrit, MCV, MCH, and MCHC but higher RBC and RDW, making red cell indices less reliable in diagnosing IDA in high‐prevalence areas [76, 77]. Transferrin saturation has shown promise as a predictor of ID, with values below 11% closely aligned with regression‐corrected definitions, although its sensitivity and specificity still vary across populations [18, 72]. The limited availability of point‐of‐care testing (POCT) in under‐resourced areas creates additional challenges, as samples often need to be sent to distant laboratories. Moreover, the use of inappropriate anticoagulants such as EDTA tubes that chelate iron and are commonly used for blood collection can make it difficult to accurately measure transferrin saturation and reducing its usefulness for diagnosis [18].

### 3.2. Systemic and Structural Barriers to ID Testing

Beyond biomarker variability and the complexity of diagnosing ID, systemic resource constraints in SSA hinder diagnostic capacity. Healthcare facilities in rural areas where ID is most prevalent lack basic laboratory infrastructure, reliable equipment, and adequately trained personnel [78, 79]. These deficits can cause delays in sample processing and compromise quality control, leading to unreliable test results. Technicians often operate with outdated equipment and face frequent shortages of reagents, which affects both accuracy and turnaround time [80]. Cost also remains a major barrier as advanced iron status tests often require expensive reagents and specialized equipment, making them unaffordable for patients and underfunded healthcare systems [72, 81].

The SSA region is known for having inadequate funding for healthcare interventions globally [82]. Health budgets are often diverted to more immediate priorities, leaving micronutrient deficiencies inadequately addressed [21]. The WHO recommends integrating iron supplementation with malaria prevention and treatment in endemic regions, but the limited access to diagnostics makes the safe implementation of targeted supplementation difficult. Also, limited knowledge and awareness about ID in SSA continue to pose major barriers to its detection, both at the healthcare provider and community levels [83]. This contributes to missed diagnoses, delayed interventions, and underestimation of the true burden in affected populations. Also, in some communities, cultural beliefs lead to these symptoms being seen as spiritual issues rather than indicators of nutritional deficiencies [84].

## 4. Innovative Approaches to Improving ID Diagnosis in SSA

The ongoing controversies surrounding accurate diagnostic methods for ID have prompted numerous recommendations to improve detection and monitoring, especially in regions with a high burden, such as SSA. In 2017, at the “Iron for Africa” workshop, which brought together experts from nine African countries, several notable interventions were proposed to enhance the health outcomes of individuals affected by ID. One key recommendation emphasized the need to improve biomarkers and develop context‐specific cut‐off values tailored to local epidemiological and clinical conditions [13]. The approaches discussed below reflect this focus and explore strategies for addressing the unique diagnostic challenges present in the SSA context.

### 4.1. Emerging Technologies for ID Testing

The diagnosis of ID has undergone major transformations in several parts of the world with the introduction of innovative technologies. These advancements have helped address long‐standing challenges associated with traditional diagnostic methods and aim to improve access to testing in resource‐limited settings. One of the most promising developments has been the emergence of POCT devices. Designed for use in field settings and remote areas lacking healthcare infrastructure, these devices offer rapid and reliable measurement of key iron biomarkers such as hemoglobin, serum ferritin, and transferrin saturation. Their performance is comparable to that of conventional laboratory techniques. In addition to speed and diagnostic accuracy, POCT devices offer a cost‐effective alternative, particularly important in SSA, where poverty often limits access to healthcare. A breakthrough in this space is the integration of mobile health applications that link POCT with smartphone‐based data collection and analysis. Some of these devices can measure iron markers such as serum ferritin using just a fingertip blood drop, while others analyze the color and features of fingernail bed photos to assess iron status, enabling real‐time diagnostics, remote monitoring, and data‐driven decision‐making [85, 86].

Recently, a highly cost‐effective ($3), portable, and easy‐to‐use smartphone‐based Hb analyzer has been developed for anemia POCT. This device has demonstrated high accuracy, sensitivity, and specificity in both clinical and home settings, making it especially suitable for use in low‐resource areas and among high‐risk populations [87]. In SSA, the HemoCue Hb301 system, which measures hemoglobin from a simple finger prick and can be easily transported to field settings, has improved access to ID diagnosis in low‐resource areas [88, 89]. It allows Hb levels to be measured at the point of care and reduces the need to rely on central laboratories, which are often inaccessible in many parts of SSA. The HemoCue Hb301 has been validated in several SSA countries and has demonstrated diagnostic accuracy similar to traditional laboratory methods, making it a practical option for use in resource‐limited settings. However, its accuracy can be influenced by factors such as how the blood sample is collected and the skill and experience of the operator. It is therefore important for stakeholders to lead validation studies’ efforts in laboratory and community settings to ensure improved performance and reliability in different settings.

The emergence of paper‐based electrochemical immunosensors designed for ferritin detection has also helped improve ID diagnostics by offering a low‐cost, sensitive, and portable solution suitable for use in low‐resource settings [90, 91]. These sensors incorporate graphene oxide–modified electrodes and covalently bound anti‐ferritin antibodies to enable sensitive and specific detection [92, 93] While not exhaustive, Table 1 summarizes POCTs for ID and assesses their feasibility in SSA. However, integrating these innovative diagnostic approaches into health systems in SSA presents several challenges. Successful adoption depends on addressing key barriers, including training healthcare workers in the correct use of new devices, ensuring regular calibration and maintenance, and tackling infrastructural issues, especially unstable electricity. Without resolving these foundational challenges, the full potential of these diagnostic tools may not be realized.

**TABLE 1:** Summary of some selected point‐of‐care tools for iron deficiency testing in SSA and their feasibility.

POCT device	Biomarker(s) measured	Advantages	Feasibility in SSA settings	Author reference
Sight OLO Hemoglobin analyzer	Hemoglobin	Fast (1 min), high sensitivity	Used in Kenyan blood banks, needs stable electricity	[94]
Masimo Pronto Plus CO‐Oximeter	Hemoglobin	No blood needed (noninvasive), quick and instant results, reusable sensor	Ideal for settings with limited lab infrastructure, ongoing studies in Ghana and other African countries	[95]
Ferritin Rapid Test Kit	Serum Ferritin	Quick, finger‐prick sample, self‐administered, low cost	Good for basic rural clinics, but ferritin is affected by inflammation	[96]
Peripheral Blood Film (PBF)	Red blood cell morphology (hypochromia, microcytosis)	Can indicate iron deficiency, low cost, and uses existing microscopes	Feasible in labs with basic microscopy, but needs trained staff	[97]
CRP Rapid Test	C‐reactive protein (CRP)	Detects inflammation, improves iron deficiency diagnosis	Used alongside ferritin to interpret results in high infection settings	[96]
sTfR Rapid Test	Soluble transferrin receptor (sTfR)	Less affected by infection than ferritin, it detects inflammation	Requires more validation, can be used in an algorithm with ferritin	[96]
Paper‐Based Colorimetric Hemoglobin Assay	Hemoglobin	Low cost, easy to use, requires no power source	Moderate accuracy, suitable for rural settings	[98]
HemoTypeSC	Hemoglobin variants (HbA, HbS, HbC)	High sensitivity, suitable for sickle cell diagnosis	Tested and deployed in many African clinics	[99]
HemeChip	Hemoglobin variants (HbA, HbS, HbC)	Automatic hemoglobin detection, portable	Robust in field settings, validated in Nigeria	[100]
Integrated MicroChip Electrophoresis	Hemoglobin variants and concentration	Simultaneous of anemia and hemoglobinopathy	Feasible and accurate in field trials	[95]

### 4.2. Establishing Population‐Based and Functionally Relevant Diagnostic Thresholds for ID Testing in SSA

Global diagnostic thresholds are important, but using regionally tailored reference ranges in SSA can improve the accuracy of laboratory interpretation and provide a clearer picture of ID in local populations. Even for the little efforts that have been achieved by some researchers on the continent to determine regionally specific reference ranges, they often remain confined to publications and rarely get adopted for use in the specific populations they studied. Immediate effort is needed to establish diagnostic thresholds tailored to the unique health, nutritional, and environmental contexts of each population across SSA. As highlighted above in the diagnostic markers and the variability in the selected thresholds for ID diagnosis, even the most sensitive biomarkers can be misleading without population‐specific reference ranges, and while some efforts have been made using appropriate statistical methods, these rarely translate into locally adopted standards. Although establishing reference ranges is generally resource‐intensive, it has the potential to improve the accurate assessment of iron status using thresholds specific to the population.

In areas where direct determination of population‐specific thresholds as recommended by organizations like the CLSI is challenging, indirect approaches using local hospital pathology records may serve as alternatives. Recent studies also suggest that functional thresholds, defined by the biomarker’s relationship to clinical outcomes like iron‐deficient erythropoiesis, may provide a more meaningful assessment [101]. For instance, pediatric studies show that ferritin centiles (2.5th percentile) often underestimate the point at which IDA develops, whereas functionally derived thresholds align more closely with the underlying physiology and disease risk [101]. The majority of efforts to derive locally relevant thresholds for various biomarkers, including red cell index markers with a notable example being the CALIPER project in Canada for the pediatric population, has improved diagnostic accuracy and clinical decision‐making in those populations [102]. Also, a study in an Emirati population recommended locally derived red cell reference ranges because of the high prevalence of α‐ and β‐thalassemia traits, which are associated with microcytosis and mildly reduced Hb levels that can complicate the interpretation of standard anemia thresholds [103]. Such hematologic variation can lead to diagnostic uncertainty when universal cut‐offs are applied without consideration of underlying population characteristics. In SSA, population studies have shown variation in Hb and hematocrit values compared with Western reference standards, suggesting that applying universal thresholds for ID may lead to diagnostic inaccuracies [104, 105]. Apart from complicating diagnosis, there is data that suggests context‐specific interactions between iron status and infection. In an African pediatric cohort, ID was associated with a reduced risk of symptomatic malaria when defined by ferritin and transferrin saturation; however, the observational design and potential confounding by exposure and immunity could rule out causal inference [106]. Unfortunately, there have been no comparable efforts in any SSA country, and this gap is especially evident for markers for ID, where locally derived thresholds are completely lacking. Locally and functionally derived thresholds are important, and in SSA, it could enhance diagnostic accuracy as it could account for local factors, improving the identification and management of both IDA and IDWA.

Table 2 compares global reference intervals for common iron biomarkers with values observed in several SSA populations. Across countries, Hb and ferritin means often fall below global thresholds [35, 46], while markers such as sTfR, transferrin saturation, and hepcidin show considerable variability due to assay differences, inflammation, infection, and genetic factors [30, 112].

**TABLE 2:** Global reference intervals versus SSA population values for iron biomarkers.

Biomarker	Global reference	SSA mean (range)	Diagnostic challenges in SSA
Hemoglobin (g/dL)	< 10.5 (6–23 mo), < 11 (24–59 mo), < 11.5 (5–11 yr), < 12 (adult women), < 13 (adult men) [108]	Kenya: 10.2 (10.1–10.3) Uganda: 10.5 (10.3–10.7) Burkina Faso: 9.6 (9.4–9.7) The Gambia: 10.7 (10.6–10.8)	Population means often below WHO thresholds; influenced by altitude, ethnicity; high prevalence of hemoglobinopathies

Serum ferritin (μg/L)	< 12 (0–5 yr), < 15 (5–8 yr) [109]	Kenya: 21.8 (20.6–23.1) Uganda: 20.8 (19.6–22) Burkina Faso: 22.2 (19.7–24.9) The Gambia: 25.1 (23.6–26.8)	Affected by inflammation; requires adjustment in malaria‐endemic regions

Soluble transferrin receptor (mg/L)	∼5–8 [110]	Kenya: 17.9 (17.5–18.3) Uganda: 6.7 (6.5–7.0) Burkina Faso: 17.7 (16.7–18.7) The Gambia: 3.4 (3.4–3.5)	Assay variability; lack of standardization across platforms in different settings

Hepcidin (μg/L)	No universal standard	Kenya: 5.8 (5.4–6.2) Uganda: 6.8 (6.4–7.2) Burkina Faso: 5.3 (4.6–6.3) The Gambia: 6 (5.3–6.7)	Limited availability; affected by diurnal variation and infection

Serum iron (μmol/L)	2.8–22.9 (< 14 yrs) [111]	Kenya: 6.5 (6.3–6.7) Uganda: NA Burkina Faso: 6 (5.7–6.4) The Gambia: 8.6 (8.3–8.9)	Diurnal variation, unreliable as a standalone test, resource constraints for proper collection

Transferrin saturation (%)	< 20 [3]	Kenya: 9.4 (9.0–9.8) Uganda: NA Burkina Faso: 8.9 (8.3–9.7) The Gambia: 12.8 (12.3–13.3)	Limited SSA data; requires multiple tests; increases cost and complexity

*Note:* Adapted from [107]. Values shown are population means with 95% confidence intervals derived from community‐based studies, not clinically defined reference intervals. As such, they are intended to illustrate population‐level differences relative to commonly used global thresholds rather than to serve as diagnostic cut‐offs. NA indicates data not available or not reported in the cited studies.

### 4.3. Community‐Based Screening and Outreach Programs

Community screening programs have proven effective in detecting various diseases in low‐resource settings, especially where access to healthcare is limited. In the context of ID detection in remote, high‐burden areas of SSA, mobile devices combined with POCT tools capable of measuring iron markers can facilitate real‐time monitoring and early diagnosis. One particularly promising approach is the use of mobile health clinics, which, though not yet widespread in SSA, have demonstrated effectiveness in delivering POCT directly to underserved populations. One of the most effective strategies is educating people about the signs and symptoms of ID through community‐based outreach programs [113]. This approach increases awareness and encourages individuals to seek regular testing when symptoms are present, ultimately improving knowledge and willingness to get tested. Caregivers can also play a key role in these efforts by recognizing early signs of ID, encouraging routine screening, and supporting treatment adherence, especially in children and other vulnerable groups.

Given the socioeconomic impact of ID in SSA, including reduced productivity, impaired cognitive and physical development, and increased healthcare costs, a multidimensional approach is needed to address ID effectively. This includes identifying high‐risk areas for targeted interventions. Such programs must be government‐funded and implemented in collaboration with civil society organizations to ensure community acceptance and that at‐risk individuals are screened and treated. For long‐term success, sustainable funding is essential, and specific targets must be set in high‐burden regions to monitor progress and maximize impact in reducing the ID burden across SSA.

### 4.4. Next‐Generation Molecular Tools for Iron Status Assessment in SSA

Molecular‐based assays extend beyond traditional diagnostic methods by providing an additional layer of diagnostic tools with high specificity and sensitivity. This allows for clearer discrimination between healthy and disease states. In SSA, their use has been limited by high costs, technical complexity, and the need for well‐resourced laboratory infrastructure. As a result, these assays are often restricted to reference laboratories, outbreak investigations, or testing for high‐risk pathogens, rather than routine clinical use. Despite these limitations, the advantages of molecular‐based approaches outweigh their drawbacks, and there is growing demand for simplified, affordable platforms that can be deployed in resource‐limited settings. In the context of ID diagnosis in SSA, molecular‐based tools such as microfluidic assays, aptamer‐ and antibody‐based platforms, and electrochemical biosensors could offer promising opportunities to improve diagnostic accuracy while reducing reagent use, sample volume, and overall cost. These approaches build on the high specificity and sensitivity of molecular detection while addressing some of the operational constraints that limit conventional laboratory testing seen in the region.

Molecular‐based assays commonly used for ferritin measurement include ELISA and automated immunoassay platforms. High‐throughput systems such as chemiluminescent and immunoturbidimetric assays are also known to provide excellent analytical performance but require costly analyzers, proprietary reagents, stable electricity, and regular maintenance. As a region that struggles with well‐resourced clinical laboratories and stable electricity, these requirements limit their feasibility. A more practical alternative is the use of manual or semi‐automated sandwich ELISA platforms. These assays require only basic laboratory infrastructure, including a microplate reader, a washer, an incubator, and standard pipettes, making them more accessible and affordable for regional‐ and district‐level laboratories [107]. Evidence supporting this approach includes the VitMin Lab ferritin sandwich ELISA, which has been widely used for over 2 decades in large‐scale micronutrient surveys. This platform has been successfully applied in several SSA countries, including The Gambia, Malawi, Côte d’Ivoire, and Cameroon, due to its low cost, small sample volume, and ability to simultaneously measure ferritin, sfTR, CRP, and alpha‐1‐acid glycoprotein [114]. While agreement for sTfR measurements has been variable, largely due to poor assay standardization across platforms, ferritin measurements have shown robust performance in low‐resource settings where this low‐cost molecular assay has been employed.

Beyond these ELISA‐based methods, there are other emerging molecular approaches that show promise for SSA. Microfluidic immunoassays allow ferritin and related biomarkers to be measured using very small blood volumes, often from finger‐prick samples, with reduced reagent consumption and shorter assay times [115]. These platforms are increasingly being designed for portability and battery operation, making them suitable for decentralized testing. Their adoption in SSA will allow clinicians in the remote areas to get an accurate information about the iron status of patients, which will improve treatments. Also, due to the cost of antibodies and lack of appropriate cold‐chain storage conditions, aptamer‐based assays, which depend on nucleic acids for detection and measurement of key biomarkers, can provide an alternative to antibodies, offering high specificity, improved thermal stability, and lower production costs.

The molecular‐based pathways provide realistic pathways to expand iron status testing in SSA and with a careful adoption and balance between analytical performance, simplicity, robustness, and affordability, they could complement existing diagnostic strategies and improve detection in the region.

## 5. Conclusion

The public health importance of ID in SSA cannot be overlooked, as it has a major impact on health and productivity. Addressing healthcare access inequities to ID management requires coordinated and sustainable efforts from government and private sector organizations. In areas with limited health facilities, innovative and cost‐effective POCTs that are portable and do not require electricity should be validated and used by community health officers to improve screening. Combining these tools with the selection of appropriate tests based on patient needs and the use of context‐specific thresholds could improve ID detection in SSA. Such efforts would ensure that even communities with minimal healthcare infrastructure have access to timely and accurate diagnosis. Achieving an ID‐free SSA will also require community‐based outreach programs to raise awareness and promote testing, using culturally sensitive approaches that foster trust and engagement. These concerted efforts could help reduce the burden of ID and support healthier, more productive populations across the region.

## Funding

No funding was received for this manuscript.

## Conflicts of Interest

The authors declare no conflicts of interest.

## Data Availability

Data sharing is not applicable to this article as no datasets were generated or analyzed during the current study.
